# Against Hierarchy: Organizing Horizontally in Healthcare

**DOI:** 10.1111/nup.70083

**Published:** 2026-04-17

**Authors:** Ryan Essex

**Affiliations:** ^1^ The George Institute for Global Health Sydney Australia; ^2^ School of Population Health UNSW Sydney Australia

**Keywords:** anarchism, authority, healthcare, hierarchy, nursing

## Abstract

Authority and hierarchy saturate social life, cutting across class, profession, gender, race, and every other line of difference. They structure what we imagine is possible, how we act, and the terms on which we do so; healthcare is no exception. From the clinical encounter to the organization of workplaces, authority is exercised, negotiated, and resisted. It shapes what is said and done in healthcare settings, who speaks, and who decides. Authority can be grounded in knowledge and experience, but it can also be conferred by gender, profession, or position. These arrangements are so entrenched that they often feel natural, almost inevitable, even when we resent them or would like to see otherwise. This article challenges that inevitability. I begin by mapping how hierarchy operates in everyday practice and asking which forms of authority, if any, should be accepted. To do so, I turn to anarchist scholarship, where authority and hierarchy have been subjected to sustained critique. I then consider the literature that documents the harms of hierarchical organization, within workplaces, but also for health more broadly. Across these domains, a consistent pattern emerges, notably that we do better when we have greater control over our lives, when we have a meaningful say in what happens around us, and when hierarchies are flattened. Finally, I show that alternatives are possible, drawing on examples of healthcare settings organized horizontally, I show that hierarchy is not inevitable nor is it desirable.

## 1 Hierarchy Is Bad for Your Health

Hierarchy is bad for your health. This claim, while perhaps provocative for some, finds substantial support across multiple bodies of evidence. Readers will likely recognize the impacts of hierarchy and authority in the workplace. Most will have seen the impact of hierarchy more generally, on friends, family, and elsewhere. Many would have submitted to arbitrary rules or procedures, even those which seemingly make little sense, while others will have felt the frustration of being told “that's just how things are.” Many will have seen the more direct harms that hierarchy perpetuates for patients and those most marginalized in society, whether this has resulted in their views and needs being overlooked or, perhaps even worse, being actively harmed because of or at the hands of authorities. Many will also have encountered authority in ways they scarcely recognize. These experiences are near‐universal because authority (and its close relative hierarchy) is pervasive; it surrounds us and shapes our lives, it shapes our workplaces, and it not only constrains our actions but also shapes how we think about the world.

At the same time, most people (including myself) at different points in our lives have exercised authority, perhaps we have found ourselves at the top of a hierarchy; perhaps we have all played our part in ensuring that some type of arbitrary policy or rule was followed. Even then, and regardless of how much or little we enjoy exercising or being subject to authority, how many people can say they can imagine a world without authority? How many people can say they can imagine healthcare without authority? This article, part provocation, part normative, seeks to elaborate on why hierarchy is detrimental to our health. Here I seek to challenge ideas about the inevitability and necessity of authority and hierarchy, not only showing that they are both unhealthy, but that things could be otherwise, when it comes to health, when it comes to how we organize healthcare and nursing. Here, I do not seek to defend hierarchy against those who insist on bureaucracy, on those who insist that hierarchy, regardless of its shortcomings, has its merits. Here, my goal is more modest: to simply show that the status quo is not desirable, nor do things have to be this way. For clarity, I am also not making a case against coordination or organization in healthcare; such an argument would, of course, be untenable. I am making the case for different forms of organizing and coordination, although what these could be are perhaps for several other papers and not something I will discuss here. Below, I will first consider the ways that authority and hierarchy operate, what they are, and how they penetrate every aspect of healthcare, from the clinical encounter to how workplaces are structured. I will then introduce the anarchist literature that has offered a basis to critique authority and hierarchy. Here, I consider the question of the forms of authority we should accept. Not only arguing that most forms of authority and hierarchy are unjust, I also move on to consider their impact on health. Here, I show that regardless of the literature, regardless of the discipline or focus, the literature all points in one direction: that we all do far better when we have control over our lives, when we are not being dominated, whether this be in the workplace or more generally. Finally, for all of those who are at least somewhat convinced but cannot imagine how things could be otherwise, I introduce some examples to show that we are not stuck, healthcare can be organized in other (non‐hierarchical) ways, with alternatives already viable.

## 2 Hierarchy in Healthcare

Before proceeding, it is necessary to clarify what I mean when I refer to authority and hierarchy, concepts that will receive considerable critical attention throughout this analysis. Authority refers to a form of power or more specifically, a form of social power that “involves the capacity of one party to exercise control over another party” (McLaughlin 2016, 54). I use the distinct but related concept, hierarchy, to refer to how authority is structured or organized. That is, hierarchy establishes authority as a norm in an organization, group, or culture; it is a type of ossified, de‐facto, or “institutionalized authority” (Amster 2018). Both concepts are related in that authority makes hierarchy possible and, in turn, hierarchy reinforces authority. But things are, perhaps unsurprisingly, more complicated than this. When it comes to authority, we can of course “be in authority, be an authority, or have authority” (Ward 2018, 43). Here we can begin to see some of the distinctions that may be drawn when it comes to authority. We might first ask why people comply, here we could draw on Weber (1946) who suggests that authority could be traditional (legitimacy via custom), charismatic (via personal qualities), or legal–rational (via rules/office/bureaucracy). While authority and hierarchy are complex and, in many ways, defy one simple explanation or framework, this paper will focus on two broad forms of authority: practical and theoretical authority (Raz 2009). Practical authority, or authority to direct conduct, refers to “the right of A to issue practical directives and the correlative duty of B to follow them or to obey them” (McLaughlin 2016, 54). Practical authority, therefore, tells you what to do and asks for your obedience (to carry out the command). This type of authority can be contrasted to theoretical authority (or epistemic authority) or authority to direct belief, that is, the “right of A to issue theoretical directives (pronouncements etc.) and the correlative duty of B to follow them—to believe or accept them” (McLaughlin 2016, 63). Theoretical authority tells you what to believe and asks for your acceptance.^1^ This could be broken down further, and to some extent, I will do this below. At the same time, however, we may also categorize both authority and hierarchy in other ways, the extent to which they are transparent or opaque, dynamic or fixed. In a more practical sense, we may also be interested in how they exist, within and between social categories and in other ways; gender, race, class, age, profession, sector, culture are all shaped by and shape authority and hierarchy, producing patterns of deference and domination, resistance and refusal (Featherstone et al. 2019; Martin and Laurin 2025). Authority and hierarchy, as I said above, are pervasive, but more than this, they are complex, relational, and context‐dependent, amongst other things.

In making sense of this in relation to nursing and healthcare, let us first consider how authority and hierarchy function in healthcare settings. Starting with the clinical encounter, we can see how authority is negotiated and resisted (Stivers and Timmermans 2020). Patients may, to varying degrees, be reliant on the theoretical knowledge of the nurse. Diagnosis may be readily accepted or even sought; it may also be resisted. The same could be said about any treatment or intervention. While most patients may be open to the advice from the nurse, others may resist, others may seek a second opinion, or even want to see a physician. The extent to which a patient is reliant will vary dependent on the nature of their complaint along with their (and the nurses) knowledge of the complaint in question. The patient may know a great deal; they may even have quite detailed ideas about what they need in terms of treatment. This may come through experience or through seeking out information themselves. Whether a patient accepts a diagnosis or recommendations for treatment may also depend on factors beyond the complaint itself, their past experiences, or beliefs about the competency of the nurse, for example. Receiving a diagnosis may also confer a degree of authority on the patient. A diagnosis may have broader social and political implications, dictating what might be expected of the patient or what they are entitled to. The patient could, of course, resist at any point (Stivers and Timmermans 2020); however, diagnosis may provide the patient with authority to make certain demands, to ask for time off work, or for exemptions from certain activities.

Expanding our focus, things become increasingly complex. Within healthcare settings, seniority, profession, gender, and ethnicity, amongst other factors, are all influential in dictating how authority is exercised and where you may sit in any given hierarchy. We see hierarchy within and between teams and between the professions; a great deal has been written on how this is negotiated between physicians and nurses (Stein 1967) and how the increasing power of management, alongside increasingly bureaucratic processes and procedures has diluted traditional forms of (mainly medical) authority (Bates and White 1961). More recent work suggests that these dynamics have not disappeared but instead persist in everyday practice, with hierarchical relations continuing to shape who is able to speak, whose concerns are taken seriously, and, in turn, what is done (Mawuena and Wilkinson 2024). Similarly, recent qualitative work has shown that hierarchies are experienced as materially consequential, limiting decision‐making power and contributing to frustration, burnout, and diminished capacity to advocate for patients, particularly among those lower in the hierarchy (Vos et al. 2026). Authority is also exercised through guidelines, processes, and procedures. Procedure is followed, when we deviate, we seek guidance, and we ask for approval from those higher up. Codes of ethics are one form of guidance that on the one hand has been seen to solidify and advance professional authority (Linker 2005). At the same time, however, such guidance could also be seen to diminish authority in taking decision making power away from individual nurses (LeFevre 2017).

Authority in healthcare must be understood as part of a broader historical and social landscape in which authority has been established, maintained, and resisted. Much has been written about medical authority, with medicine's longstanding claims of monopoly over the body and the right to treat disease and illness, including how authority has been established and maintained (Starr 2017). Not only this, we do not have to look far to see how authority has been leveraged and used to dominate and exploit, whether this be in relation to slavery and colonialism (Chakrabarti 2013; Owens 2017; Waitzkin 2015) or the oppression of transgender people (Gill‐Peterson 2018). While medicine has traditionally received the most attention in this space, other disciplines have also been critiqued. Nursing, for example, also has an authority problem. We can again see this through many historical examples, from the active complicity of nursing bodies in racial segregation under apartheid, including maintaining racially separate registers and failing to challenge the denial of care to Black patients (Esterhuizen and Van Rensburg 2021), to the involvement or nurses in the Tuskegee syphilis study (White 2023). These dynamics are not confined to the past; today, it is arguable that nursing remains “fully enclosed by the logics of late‐stage capitalism [alongside] the carceral, economic, political, and pastoral logics that structure the healthcare–industrial complex” (Dillard‐Wright and Jenkins 2024).

Authority and hierarchy are pervasive; they are also complex, dynamic, and continually exercised, negotiated, and resisted. Moreover, when we know what to look for, we can see that many of the contemporary problems faced in healthcare have to do with authority. In saying this, things do not have to be the way they are. We could (and should) do away with most forms of authority, and we could (and should) arrange our lives and our workplaces without authority and hierarchy. Here I will turn to anarchism, an ideology that offers us a particularly sharp and considered critique of authority and hierarchy.

## 3 What Anarchism Offers

If we are interested in how things could be otherwise, if we are interested in what could exist in the place of authority, anarchism provides a useful place to start. Anarchism shares substantial common ground with other radical traditions; Marxism, feminist, decolonial, and anti‐capitalist approaches all offer powerful critiques of domination and have increasingly recognized the need to challenge hierarchy in its various forms. Where these traditions differ, however, is in the scope and consistency of that critique. Anarchism is distinguished by its deep and generalized skepticism toward all authority (McLaughlin 2016), not merely specific institutions such as the state or capital, but authority in itself. Most simply, anarchism is deeply skeptical of all forms of “archy” or rule. Rather than isolating a single axis of domination, anarchism understands power as dispersed across interlocking systems, structures and ideologies. From an anarchist perspective issues such as, capitalism, patriarchalism, racism, colonialism, and others, are seen as “mutually constituting” and therefore cannot be meaningfully addressed in isolation (Rogue and Volcano 2012). For this reason, anarchism resists fixed programmes or doctrinal closure, instead embracing plurality, contestation, and ongoing revision, encouraging “an openly critical stance toward its own workings” (Amster 2018, 17). It is this reflexive and expansive critique, applied both outwardly and inwardly, that allows anarchism to remain malleable, radical, and persistently critical, even of its own processes.

For these reasons, anarchism is uniquely positioned to interrogate authority (and by implication hierarchy) in its most expansive sense. This deep skepticism toward authority provides a basis to not only think about how society should be restructured, but also how we could organize healthcare differently. Importantly (and if the above sentence is read carefully, it will be apparent that) anarchism is not the outright rejection of authority. Anarchism exercises a deep skepticism toward authority, a sort of prima facie rejection, that demands those with authority justify themselves, particularly when it comes to the exercise of power over others. Put another way, anarchism insists that “the burden of proof has to be placed on authority, and that it should be dismantled if that burden cannot be met” (Chomsky 2013). This, of course, means there are times where authority could potentially be justified. The question that naturally follows then is what types of authority should we accept? Anarchists have given this question quite a bit of thought. Many recognize the complete rejection of authority as infeasible. Take parental authority or the authority of a teacher, for example. For many anarchists, this type of authority could be justified. But what about healthcare? To get to this point, however, we first need clarity on all of the “forms” of authority I have in mind.

Consider first the theoretical authority, or the authority to direct thought or belief. Above, in my discussion about how authority exists within the context of healthcare, one of the first examples I brought up related to the clinical encounter and whether or not a patient accepts a diagnosis and or treatment recommendations. This provides an example of theoretical authority. That is, a nurse will be drawing on their knowledge to diagnose and offer an opinion, perhaps a prescription in relation to treatment. One would probably expect the patient to accept these recommendations in most cases. Here it may be noted, however, that a patient may not always accept this authority. That is, theoretical authority “does not entail recognition or enforcement” (Raz 2009, 8), so whether one is seen as an authority effectively comes down to whether one believes the expert is an authority. This, of course, comes with challenges; theoretical authority does not stem from the actual truth or falsity of what is being said; instead, the reason comes from the authority itself. That is, a nurse exercises theoretical authority when providing guidance around treatment. A patient will be asked to accept this guidance because it is coming from an expert, not because they have independently verified or disproved the content of the advice. The obvious problem is that any given theoretical authority could know very little about what they are talking about; they may have misinterpreted the evidence in question, or they may be missing critical information that may prompt them to make alternative recommendations for treatment.^2^ At the same time, the patient may dismiss these recommendations on baseless grounds, in this case dismissing the nurse's knowledge, because of some type of bias or past experience completely unrelated to what best evidence may dictate. Here, the difficulty comes in striking a balance between openness and skepticism toward theoretical authority. Anarchist thinkers have a range of responses to theoretical authority. Some accept the idea of theoretical authority. On face value, it is sensible to defer to those more knowledgeable than us when it comes to certain things. At the same time, however, complete deference is problematic. This qualified acceptance, or cautious skepticism toward theoretical authority, is perhaps best reflected in this widely quoted passage from Bakunin (1882) who asserted:

In the matter of boots, I refer to the authority of the bootmaker; concerning houses, canals, or railroads, I consult that of the architect or engineer. For such or such special knowledge, I apply to such or such a savant. But I allow neither the bootmaker nor the architect nor the savant to impose his authority upon me. I listen to them freely and with all the respect merited by their intelligence, their character, and their knowledge, reserving always my incontestable right of criticism and censure.

While the bootmaker in this case was not attempting to impose his authority, one further problem is that the line between theoretical and practical authority is not always clear, particularly in healthcare. Within any given healthcare organization, there will be multiple specialized teams. Within each of these teams, there will presumably be some junior, some more senior staff, all with varying levels of experience and knowledge. In thinking about theoretical authority in this situation, it would seem, on face value, that it may be the right thing to do to defer to those more knowledgeable when it comes to certain issues. However, one problem here is that arguments for practical authority often come from theoretical authority (Raz 2009). That is, if someone has legitimate expertise in a certain field, they might claim not just a right to be believed (theoretical authority) but also a right to be obeyed (practical authority).^3^ This is often the case in healthcare, with healthcare workers often authorities not “only on the causes of illness but also on their cures” (Raz 2009, 8). Anarchists would be skeptical of such a leap, recognizing that superior knowledge or skill does not entitle another to command or direct another person's actions.

In addition to being difficult to separate, theoretical and practical authority come in a range of forms or types: political authority, parental authority, spontaneous authority, economic authority, and managerial authority, to name just a few. I could go on, but I will limit the discussion to a few particularly pervasive forms of practical authority found throughout many healthcare settings. Managerial authority warrants particular attention. We have all experienced managerial authority, or authority that involves “the right of certain individuals to issue directives and to have them followed based on their position (or *who* they are) *within* an organizational hierarchy” (McLaughlin 2016, 71). Managerial authority, like its close relative bureaucratic authority, has an “emphasis on *office*” and while it may be more “personal and unofficial” than bureaucratic forms of authority, this authority does not rely on the pronouncement or message itself, even if a manager is correct, you do not comply because of the content of the message or pronouncement, but because the message has come from your manager. Anarchists would almost always reject such authority. In addition, many would also point to some of the issues created by such authority, issues that many of us are likely familiar with. One notable issue relates to the way that hierarchy in this form closes feedback loops, ensuring communication only travels one way through organizations. Carson (2020, 367), for example, explains how this one‐way communication results in several further and compounding issues in organizations, with this only re‐inforced by the fact that “senior management … don't live under the effects of their policy, and they are insulated from negative feedback.” Over time, this creates an increasing conflict of interest between those at either end of the hierarchy. That is, because of this disconnect, those on the front lines (or at the bottom of the hierarchy) often have little connection with the overall goals of the organization, creating a situation where “workers have absolutely no incentive to contribute their judgment to improving work processes, and every incentive to sabotage efficiency.” This is perhaps best summed up by Ward (2018, 43) who explains:

The fantastic inefficiency of any hierarchical organization—any factory, office, university, warehouse, or hospital—is the outcome of two almost invariable characteristics. One is that the knowledge and wisdom of the people at the bottom of the pyramid find no place in the decision‐making leadership hierarchy of the institution. Frequently, it is devoted to making the institution work in spite of the formal leadership structure, or alternatively to sabotaging the ostensible function of the institution, because it is none of their choosing. The other is that they would rather not be there anyway: they are there through economic necessity rather than through identification with a common task which throws up its own shifting and functional leadership.

One final form of practical authority that is more palatable for anarchists is spontaneous authority, or authority that involves “the immediate assumption of authority in an emergency situation” (McLaughlin 2016, 70). One scenario I didn't discuss above relates to emergencies, something which are fairly commonplace in healthcare settings, and something most nurses would be largely trained to deal with. When it comes to an emergency related to a patient, in many cases, there will be clear guidelines and or a structure that is dictated by profession or seniority, for example. This may not always be the case, however, and at other times, roles and responses to the emergency in question may be far less clear. In thinking about whether we should defer in this case and who we should defer to, it is worth thinking about some of the other features of authority, notably whether it is voluntary and temporary, that is, whether we are voluntarily deferring or submitting, and whether this will be temporary. We can see that any response in an emergency will likely fit such criteria. Because of this, for many anarchists, such authority may be palatable when it comes to emergencies, as long as this does not become a permanent or ongoing arrangement. This idea around authority being temporary and voluntary could also lead us to accept forms of authority when it comes to routine encounters between nurses and patients, with it possible that authority, both theoretical and practical, may be largely exercised voluntarily and for a limited time.

While the issue of authority has occupied a great deal of anarchist thought, anarchism cannot be reduced to a preoccupation with this single issue. It has also given considerable attention to what should exist as an alternative. In place of structures and systems that rely on authority and hierarchy, anarchism proposes alternative modes of organizing. Anarchist thought has long emphasized practices such as mutual aid, horizontal decision‐making and decentralized coordination, forms of organization that prioritize cooperation, reciprocity, and collective responsibility over command and control (Graeber 2004). Another central concept is prefiguration, that is, the attempt to build, in the present, the kinds of social relations and institutions we wish to see in the future (Boggs 1977). These principles, alongside a deep skepticism toward authority, guide anarchist organizing, with horizontality, care, and cooperation positioned as alternatives to hierarchy, domination, and control. In this respect, anarchism is also about building, imagining, and it also provides tools to organize in the here and now, to navigate and negotiate our present while experimenting with alternatives. It is not, as many would have you believe, synonymous with chaos and disorder.^4^ Whether through mutual aid, DIY practices, or efforts to support the health of refugees and migrants, anarchist practices have long been concerned with health, well‐being, and human flourishing as much as they have been critical of existing institutions, systems, and organization (Essex 2023; Scott 2018; Shantz 2020).

The above discussion only scratches the surface when it comes to anarchist critiques of authority, even in only considering managerial authority and theoretical and spontaneous authority, along with their rejection and qualified acceptance, we can begin to imagine the extent to which current arrangements would have to transform if we were to organize them along anarchist lines. While this deep skepticism toward authority and rejection of hierarchy in all forms is a core and distinguishing characteristic of anarchism, these ideas are not foreign to other ideological approaches, nor have they been missed by those with an interest in health. One example here comes from the global health literature. Consider this quote from two well‐known global health researchers, who have not, to my knowledge, (publicly) identified as anarchists:

To decolonize global health is to remove all forms of supremacy within all spaces of global health practice, within countries, between countries, and at the global level. Supremacy is not restricted to White supremacy or male domination. It concerns what happens not only between people from [high‐income countries and low and middle‐income countries] HICs and LMICs but also what happens between groups and individuals within HICs and within LMICs. Supremacy is there, glaringly, in how global health organizations operate, who runs them, where they are located, who holds the purse strings, who sets the agenda, and whose views, histories, and knowledge are taken seriously. Supremacy is seen in a persistent disregard for local and Indigenous knowledge, pretense of knowledge, refusal to learn from places and people too often deemed “inferior,” and failure to see that there are many ways of being and doing. Supremacy is there in persisting colonial and imperialist (European and otherwise) attitudes, in stark and disguised racism, White supremacy, White saviourism, and displays of class, caste, religious, and ethnic superiority, in the acquiescing tolerance for extractive capitalism, patriarchy, and much more (Abimbola and Pai 2020, 1627).

While I acknowledge many readers may not consider themselves anarchists, and even if sympathetic to my argument above, it may still be difficult to imagine healthcare without authority. Even for those less than convinced, I would invite considerations of what could be gained by having a far more attuned approach to authority, in critiquing the purpose that authority serves, whether it is necessary, and how things could be done differently. I would also ask readers to think about what would happen if we changed the burden of proof. Most readers would likely agree that, for the most part, we could shed vast amounts of red tape, bureaucracy, and flatten hierarchies in healthcare, and perhaps this could help us get back to what nurses and other healthcare workers (should) actually do, care for people. Below I want to offer two more reasons why we should adopt a far more skeptical stance toward authority in healthcare, one offering a more negative case, discussing the impacts of hierarchy, with a focus on health and well‐being, and one more positive and hopeful, showing that things don't have to be the way they are, that is, there are already examples of horizontal organizing that we can draw upon in shaping healthcare along different lines.

## 4 Hierarchy and Its Impact on Health

Above, I have introduced how anarchism has approached authority and hierarchy. This approach is best described as one of great skepticism that demands quite a bit from authority if it is to be justified. I also briefly touched upon some of the other knock‐on effects of authority in organizations, in how it creates a one‐way channel of communication and creates divergent goals for those at different levels of the hierarchy. Here, I want to focus on some other reasons why we should reject authority, namely, its impact on health and well‐being. My argument here is a relatively simple one. Hierarchy is bad for health because it tends to reduce people's control over the conditions of their lives, suppress their capacity to influence what happens around them, while also entrenching unequal social relations. These are not separate issues; they all boil down to authority and hierarchy. They are also closely related and often mutually reinforcing. Where hierarchy is present, voice is often constrained, autonomy is diminished, and inequality is reproduced; when this is experienced with greater intensity health tends to suffer. Conversely, where people have greater control over their lives, where decision‐making is more democratic, and where social relations are more equal, health and well‐being tend to improve. The relationship between hierarchy and health can be seen across several bodies of literature. In some cases, the connection is quite direct, as in studies examining the effects of rank, control, or inequality on mortality and morbidity (Bosma et al. 1997; Marmot et al. 1991, 1978). In others, it is more indirect, visible in the way hierarchy shapes working conditions, access to resources, exposure to stress (Beckie 2012; Guidi et al. 2020), or the extent to which people are able to participate in decisions affecting their lives (Elovainio et al. 2002; Robbins et al. 2012). While this literature does not always speak in the language of authority, they consistently point toward the same conclusion, notably that health is shaped by the degree of control, dignity, equality, and collective say that people are able to exercise in the worlds they inhabit.

With this, and even looking only at healthcare, I run into the problem of where to start. Should I start with the issues of communication within healthcare settings, or perhaps the role that hierarchy plays in ensuring patient preferences are routinely overlooked, or why miscommunication occurs so regularly in surgery, or perhaps why bullying and harassment are so widespread? On this, there has been a small but growing body of evidence that has examined the impact of hierarchy in healthcare settings, a body of work that speaks to all of these issues (Essex, Kennedy, et al. 2023; Kim et al. 2020; Okuyama et al. 2014). One of the problems when we look to this literature however is that any examination of authority is likely to underestimate its impact, often we see studies that examine teamwork, communication, or resistance, all of which are influenced and shaped by authority and hierarchy, even when not the subject of the study. It is a notoriously difficult thing to examine in organizations for this reason, because, as I said above, authority and hierarchy are pervasive. In saying this, there is a vast literature that gives insight into the likely mechanisms through which hierarchy shapes health. Again, while often using different terminology, there is substantial evidence that speaks to how sitting lower in a hierarchy generates chronic stress and physiological dysregulation (Parker et al. 2022) by limiting autonomy (Bosma et al. 1997; Marmot 2006), by normalizing unequal treatment and organizational injustice (Elovainio et al. 2010; Robbins et al. 2012), by silencing concerns (Delpino et al. 2023), and by closing down opportunities for collective action or meaningful participation (Orton et al. 2019). In healthcare settings, the literature suggests burnout, frustration, patient harm, and poor communication are all symptoms of authority and hierarchy (Peter et al. 2020; Vos et al. 2026).

There are several further notable studies worth mentioning. Consider a study published in *The Lancet* by Bollyky et al. (2019) that used data spanning 170 countries. This study not only sought to assess the association between democracy and mortality, but also explored the pathways that explained differences in health status and mortality. This study found that people in democracies, as opposed to autocracies, were more likely to have better health, with the more democratic a country was, the better the health of its citizens. The authors go on to consider why this was the case. One of the first reasons had to do with accountability of government in that “democracies should have a greater incentive than autocracies to provide health‐promoting resources and services to a larger proportion of the population.” The next reason, they speculate, had to do with the fact that “[d]democracies are more open to feedback from a broader range of interest groups, more protective of media freedom, and might be more willing to use that feedback to improve their public health programs” (p. 1629). In other words, people that have a greater say in their lives and how they are governed also generally have better health. Put another way, when people have more capacity to shape the conditions under which they live, health tends to improve.

When it comes to life expectancy and mortality, we see the influence of other factors as well, beyond the political systems in which people live. Over 40 years ago, Caldwell (1986) published an influential study that sought to identify why some regions, despite still having few resources, did relatively well when it came to mortality. This study examined the success of three countries (China, Costa Rica, and Sri Lanka) and one Indian state (Kerala) that achieved exceptional success in lowering mortality despite having limited financial resources. The study challenged the assumption at the time that greater access to resources, financial or otherwise, led to better health. Identified in the success in these regions were a number of common features, including “a substantial degree of female autonomy, a dedication to education, an open political system, a largely civilian society without a rigid class structure, a history of egalitarianism and radicalism and of national consensus arising from political contest with marked elements of populism” (Caldwell 1986). We see this play out elsewhere and in other ways. Take the case of Porto Alegre, a city in southern Brazil. In 1989, the city introduced a series of reforms that were aimed at improving living standards and tackling inequality. One of these reforms was participatory budgeting (orçamento participativo), which did what might be expected: gave residents a say in how city funds were spent. After some trouble getting this initiative off the ground, by the early 1990s there was widespread participation, and by 2000, there were upwards of 14,000 people attending the first assembly of the year (Baiocchi 2005). This initiative led to substantial improvement in the city's poorer areas, including greater investment in infrastructure. Sewage and water infrastructure was expanded and covered the entire city, while there was a threefold increase in the number of children attending school. Health spending also went up, and administrative spending went down over this period (Baiocchi 2005). While some studies suggested no impacts on well‐being, despite this increased spending (Boulding and Wampler 2010), other studies suggest a more direct impact on health as well with a significant reduction in the infant mortality rates among municipalities that adopted participatory budgeting (Gonçalves 2014). Together, these studies show that health is shaped not only by what people have, or socioeconomic status, for example, but by the social relations through which life is organized. That is, ordinary people had greater influence over collective decisions, with budgeting redistributed both resources and voice.

The above studies sit within a broader public health literature, which is clear in its message, notably that equality is far better for your health, regardless of where you sit in the social hierarchy (Schrecker and Bambra 2015; Wilkinson and Pickett 2010). This is another point worth dwelling on. More unequal societies tend to be marked by greater stress, insecurity, and exclusion, particularly felt by those at the bottom of hierarchies, whether these have to do with gender, profession, or class, to name just a few. The literature here overwhelmingly suggests we do better, all of us, when resources and decision‐making are more equally distributed. Alongside this literature that warns against some of the more general harms of hierarchy sits a literature that many readers will be familiar with, a literature that speaks to the harms of more specific forms of authority and hierarchy, whether this be racism (Krieger 2014) or hierarchy based around citizenship (Dubal et al. 2021). Racism, bordering practices, and other forms of social subordination are hierarchical arrangements that distribute vulnerability, premature death, and suffering along lines of power.

We see this play out elsewhere, even when isolating our analysis to the workplace. Here, we cannot go past the Whitehall studies. The first of these studies was carried out in the United Kingdom and followed over 17,000 male civil servants over a period of 10 years starting in 1967, examining cardiovascular disease and mortality rates. The results of this study were quite telling. Being employed in a lower grade was associated with an increased number of risk factors, like obesity, smoking, and less physical activity; it was also associated with higher levels of mortality. What is particularly striking is that even after controlling for these risk factors, those in lower grades were still twice as likely to die from coronary heart disease (Marmot et al. 1978). A second study, carried out between 1985 and 1988 virtually replicated these findings, again suggesting that civil servants in lower grades had higher morbidity, which was not fully explained by any additional risk factors that were present (Marmot et al. 1991). The enduring significance of the Whitehall studies lies in what they suggest about rank and control. Health did not simply map onto socioeconomic status, rather it was patterned by social hierarchy, with those lower having worse health outcomes.

Taken together, this literature points in one direction. Hierarchy is harmful, not only when it is overtly abusive, extreme, or obviously unjust. It is harmful more routinely and more quietly, through the ordinary organization of social life. While further evidence could be listed here, the point has been sufficiently established. Across time and place, in large‐ and small‐scale studies, within workplaces and beyond them, the pattern is strikingly consistent, namely, we do far better when we contend with less hierarchy and authority; when things are fair, when we have choice and control in our lives, we are generally better off, in a general sense and when it comes to our health. Where authority concentrates control, closes down participation, and entrenches subordination, health is likely to worsen. What this literature points to, overwhelmingly, whether in the workplace or elsewhere, we do better without authority, we do better without hierarchy. We are healthier, as are those around us. I have not come across any studies, despite searching, that speak to the virtues of authority and hierarchy when it comes to health and well‐being. When it comes to good health, the less authority the better.

## 5 Healthcare Without Hierarchy

Things don't have to be how they are. We do not have to (begrudgingly) accept hierarchy or authority in healthcare, or their associated harms. Nor is it a good reason to submit because we simply cannot imagine how things could be otherwise. Here I want to address this final point. We can and should organize differently; we can and should organize horizontally, and importantly, we do not have to look very far to find inspiration here. These are not just possibilities, but actualities, with several examples past and present that speak to how things could be otherwise.

Starting with the everyday and outside of healthcare settings, we find such forms of organizing everywhere, day to day, in seemingly mundane activities, things like carpooling, social clubs, sharing a meal, or borrowing a ladder from your neighbor, are all examples of organization and action that entail little authority. Any decision made by consensus. People share, give, and receive without any expectation of profit, obligation, or control. I said earlier that authority and hierarchy are pervasive, they are, but they are not all encompassing, when we know where to look evidence for another way of organizing is already here, hidden in plain sight. These everyday practices are important because they demonstrate that coordination does not require hierarchy. They show that people are capable of organizing collectively, making decisions together, and meeting shared needs without recourse to authority. Even in more elaborate systems, we can see how things could be different. On this point, Ward (2018, 53) asks us to think about the railways and how international post works:

You can post a letter from here to China or confident that it will arrive, as a result of freely arrived at agreements between different national post offices, without there being any central world postal authority at all. Or you can travel across Europe over the lines of a dozen railway systems—capitalist and communist—co‐ordinated by agreement between different undertakings, without any kind of central railway authority. The same is true of broadcasting organizations and several other kinds of internationally co‐ordinated activities.

When it comes to healthcare, perhaps one of the most famous examples of how we could do things differently comes from the UK and the Peckham experiment. Set up in 1926 by physicians George Scott Williamson and Innes Hope Pearse, the project challenged the prevailing idea that health was simply the absence of illness. It was based in the Pioneer Health Centre, a building designed to function as a kind of family club. Families joined by paying a small fee and agreeing to an annual “health overhaul,” a comprehensive check‐up. At its peak, nearly 850 families were involved, with hundreds using the center each day (Charkin 2014). The services and activities were wide‐ranging, including a swimming pool, gymnasium, cafeteria, nursery, and spaces for games and leisure. What made the centre distinctive, however, was not the infrastructure, but its ethos. There were no schedules, no classes, no managers setting the pace or rules. The philosophy was one of non‐interference: “there is nothing to tell the members what to do, when to do it or how to do it” (Pioneer Health Center 1930). While many would expect chaos under such conditions, and while at first there was, over time what emerged was an “astounding sense of order and ease… an inherent vitality in this community of families representing all age groups, many backgrounds and varying degrees of skill and competence” (Pearse and Crocker 1941). People flourished organically, and a range of activities began to emerge: music, sports, debates, performances, and social gatherings. Members often reported better health and a stronger engagement in their own well‐being. The Peckham experiment suggested that without oversight, regulation, or expert direction, people were more than capable of cultivating their own health and creating a cooperative, caring community.

More recent illustrations can be found in Greece, where over the past two decades close to 100 solidarity clinics have been established (Evlampidou and Kogevinas 2019). Their growth was tied to the economic crash of 2008, which triggered a cascade of austerity measures, which pushed unemployment to record levels while gutting social and health services. By 2011, cuts to hospital budgets had produced widespread shortages and severely restricted access to care. It was estimated that around 30% of the population, along with hundreds of thousands of undocumented migrants, were excluded from health and social security system (Kogevinas 2018). Into this vacuum stepped grassroots initiatives, with solidarity clinics springing up across the country (Malamidis 2020). These clinics had no central authority; each was shaped by local circumstances, run collectively by volunteers, and provided care without a charge. Some of the largest clinics serve more than 500 uninsured or partially insured patients every month, offering a mix of primary and preventive care (Evlampidou and Kogevinas 2019). Alongside the clinics, other grassroots projects flourished, community kitchens, cultural centers, and worker‐run spaces. One illustrative case was the occupation of the abandoned Vio.Me tile factory in Thessaloniki in 2013. Workers took over and converted it into a cooperative producing ecological cleaning products. Two years later, they set up a health clinic inside the occupied site. This Workers' Health Centre inside Vio.Me represented more than a place of treatment. Patients were not called patients at all, but “incomers,” with empowerment treated as the first step of care. Its aim was to dismantle the hierarchies inherent in medical practice, to undo “the subordination they have experienced as a person who has been seen either as a body, or a worker, or a person—but not all three at once” (Thornton 2020). What was most striking in this clinic and many others, was not the fact that so many people had volunteered their time, or the fact they were offering their expertise free of change, but the provision of empathy, connection, and recognition in the midst of a system that had abandoned them (Thornton 2020). Like other solidarity clinics, the Workers' Health Centre deliberately positioned itself outside the control of the state, charity, or philanthropic donors (CareNotes Collective 2020). These spaces, while essential for plugging the gaps left by austerity, were also building, prefiguring a different form of care, one that resisted medical authority, rejected capitalist co‐optation, and sought to create forms of health practice rooted in solidarity, autonomy, and mutual aid.

While these examples emerge from crisis and collective struggle, alternative forms of organizing are not limited to such contexts. Buurtzorg (which means “neighborhood care”) is a home care organization based in the Netherlands. Founded almost 20 years ago, the organization is structured around small, autonomous teams of nurses. These teams, of 10−12 nurses, handle a local neighborhood (buurt), and a group of patients, taking care of everything themselves, from clinical tasks to scheduling to administrative work. There are no intermediaries, with teams operating virtually autonomously, that is, teams have “complete autonomy to carry out their work as they see fit—no targets, no allocated time slots per patient, no tick‐box care” (Upson 2020). In 2015, there were close to 700 teams operating throughout the Netherlands. In terms of administrative structures, only a small back office exists, with about 50 staff who handle things like salary and other administrative tasks (Gray et al. 2015). There have been several other advantages of this approach, most notably, high staff and patient satisfaction and reduced costs related to care (Gray et al. 2015). Buurtzorg is particularly instructive, showing how horizontal organizing can work in more routine healthcare settings. It shows how autonomy, trust, and decentralization can produce not only more satisfying work environments but also improved patient outcomes and reduced costs. Here, hierarchy is replaced with a different form of coordination, one based on professional judgment, collaboration, and shared responsibility.

While vastly different, and while each example I provided has a very different approach toward authority, together they show that organizing things differently, even in the deeply imperfect present, is not beyond reach. That is, rather than being exceptional, these cases point to possibilities that already exist, but are often overlooked or dismissed. Taken together, they also illustrate that healthcare does not have to be organized through rigid hierarchies, top‐down control, or managerial authority. Alternative arrangements, grounded in mutual aid, autonomy, and collective decision‐making, are not only imaginable, but already in practice.

## 6 Conclusion

Returning to my central point, hierarchy is bad for our health; fortunately, however, things do not have to be the way they are. Authority and hierarchy are not inevitable, nor are they necessary. We do better, patients do better, society does better when authority is questioned, when control is shared, and when decisions are made collectively. Whether in everyday, seemingly mundane practices or in more deliberate efforts, we see that healthcare can be organized horizontally, without managers, without rigid chains of command, without the assumption that expertise entitles one to rule. There are a number of examples, these and many others, that provide us with viable blueprints of how things may be otherwise. We cannot keep excusing authority as inevitable; our health depends on building systems where care, not control, takes precedence.

## Funding

The author has nothing to report.

## Ethics Statement

The author has nothing to report.

## Conflicts of Interest

The author declares no conflicts of interest.

## Data Availability

The author has nothing to report.

## Endnotes

1 McLaughlin also speaks about moral authority or as “the right to issue theoretical directives with practical force or moral principles that govern practice (the practice of another).” I would direct readers here if interested; however, in the interests of space and scope, it is not a form of authority I will consider here.

2 There is also of course a broader literature that is critical of diagnosis itself. This is relevant here, but beyond the scope of this article.

3 Thinking back to executive and non‐executive authority as discussed above, De George, R. T. (1985). *The Nature and Limits of Authority*. University Press of Kansas. provides this example that speaks to this point: “The informal patient‐doctor relation assumes that both doctor and patient are interested in the patient's health and that the patient goes to the doctor for advice and instruction. The doctor is an epistemic authority who has no right to command… Yet patients frequently react to the prescriptions of a doctor as to someone who holds imperative authority; and in these cases, we can say that the doctor is a de facto executive authority with respect to such patients.”

4 I would direct readers to McLaughlin (2016). *Anarchism and authority: A philosophical introduction to classical anarchism*. Routledge, for a lengthier discussion about critiques/misconceptions in relation to anarchism.
